# Iron Deficiency and Iron Deficiency Anemia in Women with and without Obesity: NHANES 2001–2006

**DOI:** 10.3390/nu15102272

**Published:** 2023-05-11

**Authors:** Sixtus Aguree, Arthur Owora, Misty Hawkins, Manju B. Reddy

**Affiliations:** 1 Department of Applied Health Science, School of Public Health-Bloomington, Indiana University, Bloomington, IN 47405, USA; 2 Department of Pediatrics, School of Medicine, Indiana University, Indianapolis, IN 47405, USA; 3 Department of Health and Wellness Design, School of Public Health, Indiana University, Bloomington, IN 47405, USA; 4 Department of Food Science and Human Nutrition, Iowa State University, Ames, IA 50011, USA

**Keywords:** iron deficiency, ferritin, transferrin saturation, transferrin, anemia, hemoglobin, obesity, overweight

## Abstract

Obesity has been linked to numerous health and nutritional problems, including impaired iron metabolism, a common cause of anemia. We aimed to determine the prevalence of anemia, iron deficiency (ID), and iron deficiency anemia (IDA) among women aged 20–49 years based on body mass index (BMI) status. We used measures of iron status and body mass index from the 2001–2006 National Health and Nutrition Examination Survey (NHANES). Mean serum ferritin, erythrocyte protoporphyrin, and soluble transferrin receptor were higher, while those of serum iron, percent transferrin saturation, and mean cell volume (MCV) were lower in women with obesity than those with normal weight (all *p* < 0.016). ID based on the ferritin model was 12.5 ± 1.0% vs. 22.9 ± 1.6% (*p* < 0.001); 9.0 ± 0.9% vs. 20.0 ± 1.3% (*p* < 0.001) based on the MCV model; and 8.1 ± 1.0% vs. 10.5 ± 1.2% (*p* > 0.05) based on the BII model for women with normal weight and women with obesity, respectively. Anemia prevalence was 5.5 ± 0.8% (normal) vs. 9.3 ± 1.0% (obese) (*p* = 0.005). The IDA estimates based on the ferritin and MCV models were similar but higher than that from the BII model (*p* < 0.001). Generally, the prevalence rates of ID and anemia (and IDA) were higher for women with obesity, but the method used to define deficiency mattered. The choice of iron indices is important for estimating ID and IDA in populations with obesity.

## 1. Introduction

Obesity is a public health problem in many countries worldwide [1,2], with a prevalence of 42% among adults in the United States [3]. A rising obesity rate has significant implications for iron deficiency (ID) because obesity-associated low-grade inflammation (subclinical level of proinflammatory activity) [4,5,6,7] impairs iron homeostasis. This impairment results in a low circulating serum iron level and reduced iron absorption and storage, or ID [8,9,10,11]. ID is the leading cause of anemia, a condition characterized by low blood concentration of hemoglobin, and accounts for the majority of the diagnosed anemia cases [12,13] (i.e., iron deficiency anemia; IDA). Low iron status and associated IDA are linked to poorer biopsychosocial and neurological health outcomes and functional impairment, given the constellation of common symptoms, such as dyspnea, fatigue, tachycardia, and syncope [14,15]. ID disproportionately affects preschoolers and women of childbearing age [12,16]. For example, in the US, 15% of children aged 12–23 months are iron deficient, while 10% of females experience iron deficiency [17,18]. The disproportionate rates of ID in children and women align with elevated rates of severe obesity in these populations. Severe pediatric obesity rates have escalated since the 1980s [19], and women with severe obesity (11.5%) currently outnumber men (6.9%) in the United States [20]. Given the rising levels of obesity in these groups, particularly severe obesity, and its impact on iron metabolism and status, studies that focus on providing how iron biomarkers and ID vary by BMI in large populations could be important for public health education and policy. Such studies should also account for the various methods used for assessing iron status, given that subgroup meta-analytic results indicate that the method used to classify ID impacts whether associations with obesity are detected [21].

As alluded to above, assessing iron status in a population involves the combination of several iron biomarkers to classify deficiency or abnormal iron status. A combination of two or more iron indices is recommended when assessing ID [22]. Commonly used indicators include serum ferritin (SF), serum iron (SI), total iron-binding capacity (TIBC), transferrin saturation (TS) (TS (%) = [Fe/TIBC] × 100) [23], soluble transferrin receptor (sTfR), erythrocyte protoporphyrin (EP), and mean corpuscular volume (MCV) and hemoglobin (Hb) [24]. These iron status indicators and their relation to ID have been discussed elsewhere [25,26]. The three models most commonly used to define ID at the population level are the ferritin, MCV, and total body iron (hereafter, body iron index or BII) models. Individuals are classified as iron deficient when abnormal values are present in at least two of the three measures of SF, TS, and EP (the ferritin model) [27,28]^,^ or MCV, TS, and EP (the MCV model) [28,29]. Cook and colleagues introduced the BII model as an indicator of iron stores [30,31] calculated as the log sTfR to SF ratio, where BII < 0 mg/kg is considered iron deficient [30,31]. According to Cogswell et al. [32], the BII model has been adopted to assess ID among NHANES participants [32,33] and to monitor ID in the US population [34,35]. Before the adoption of the BII model by the NHANES, the ferritin model was used for the same purpose [25,27,29]. The MCV model has also been widely used to estimate ID in previous NHANES data [36,37]. The prevalence of ID and IDA estimated by the ferritin model and the BII model vary considerably [32,38]. For example, using the NHANES 2003–2006 data, Cogswell et al. [32] compared the prevalence of ID in children and nonpregnant females in the US via the body iron model [30,31] and the ferritin model [27,28]. They found that estimates of ID prevalence were higher using the ferritin model (15.7% vs. the BII model (9.2%) for females aged 20–49. Similar findings were reported by Sekhar et al. for adolescent females; ID prevalence for the ferritin model was 13.1%, and it was 9.1% based on the BII model [38].

Despite evidence suggesting that ID and obesity are linked, population-based studies comparing ID, anemia, and IDA prevalence across BMI categories are limited. This analysis presents the results on how different iron biomarker concentrations differ by BMI status in women aged 20–49 years. Similarly, the prevalence of ID, anemia, and IDA by BMI categories are also presented. We hypothesized that the three models would provide different prevalence estimates across BMI categories, particularly for the obese category, and could provide insight when interpreting results of ID data from different studies or combining and interpreting ID estimates in meta-analysis.

## 2. Materials and Methods

### 2.1. Study Design, Participants, and Setting

This study used data from the 2001–2006 National Health and Nutrition Examination Survey (NHANES). Data from the NHANES were from a cross-sectional survey that used a stratified multistage probability design to obtain a nationally representative sample of the civilian, non-institutionalized US populations. All NHANES data were collected by the National Center for Health Statistics of the Centers for Disease Control and Prevention. The NHANES survey protocol was approved by the Research Ethics Review Board at the National Center for Health Statistics, and written informed consent was obtained from all participants or their proxies. A description of the procedures for collecting the NHANES data can be found on the Centers for Disease Control and Prevention website and elsewhere (Available from http://www.cdc.gov/nchs/nhanes.htm (accessed on 25 October 2022)) [39,40]. The NHANES protocol includes an in-home interview, a health examination at a mobile examination center, and a follow-up telephone call about 3–10 days after the examination. Demographic data, including age, gender, education, race and Hispanic origin, and family poverty-to-income ratio were collected via a computer-assisted software during the home interview. At the mobile examination center, the participants underwent a health examination and anthropometric measurements (e.g., height and weight), and their biological specimens were also collected for laboratory assessment.

This analysis was completed using 6 data cycles from the NHANES 2001–2002, 2003–2004, and 2005–2006 to improve the reliability and stability of statistical estimates. Review by the institutional review board was not required for the current analysis because the NHANES data survey protocol had been approved, and the data allow public access. The analytic sample included women aged ≥20–49 y who were not pregnant or breastfeeding at the time of participation and with no missing ferritin, MCV, and hemoglobin data. We excluded women with an infection (white blood cell counts > 10.0 × 10^9^/L) and those with possible liver disease because liver disease and infection can influence biochemical indices [41]. We defined abnormal liver enzyme levels as at least 1 of 2 abnormal elevations of alanine aminotransferase (ALT > 70 U/L) and aspartate aminotransferase (AST > 70 U/L)) [42]. CRP concentrations >6.0 mg/L were deemed indicative of inflammation [32] and were excluded. Women with diabetes were also excluded. After the exclusion criteria, the available sample size was 2514 subjects.

In this analysis, we categorized the demographic variables as follows: age groups (20–34 and 35–49 y), race and Hispanic origin (Non-Hispanic White, Non-Hispanic Black, Mexican American or other Hispanic, and “other” (referred to other race including multi-racial)), and education (less than high school, high school diploma/GED or equivalent, completed some college or obtained an associate’s degree, and bachelor’s degree or more). The family poverty-to-income ratio was categorized into three groups, namely 0.00—130%, >130–350%, and >350%, because a family poverty-to-income ratio of 130% is the cutoff to determine financial eligibility for the Supplemental Nutrition Assistance Program(SNAP) [39]. Body mass index (BMI), obtained from the height and weight measurements collected at the mobile examination center, was treated as a categorical variable: BMI of <18.5 indicates underweight, 18.5– < 25 indicates normal, 25– < 30 indicates overweight, and ≥30 kg/m^2^ indicates obese [43,44].

### 2.2. Laboratory Methods

The hematologic (hemoglobin and MCV) and biochemical (SF, TS, SI, EPP, and sTfR) tests were measured in the NHANES laboratory at the CDC. The details of the assay methods for these laboratory parameters are available elsewhere [29,32,33,45,46]. Briefly, hemoglobin and MCV were measured as part of complete blood count parameters at the mobile examination centers using the Beckman Coulter MAXM hematology flow cytometer (Beckman Coulter, Brea, CA, USA, Beckman Coulter Inc., Fullerton, CA, USA). Two methods were used to measure serum ferritin and sTfR. A single-incubation 2-site immunoradiometric assay (BioRad Laboratories, Hercules, CA, USA) was used in 2003. As of 2004, ferritin and sTfR were measured on a Hitachi 912 clinical analyzer (Roche Diagnostics, Mannheim, Germany) using the Roche Tina-quant immunoturbidimetric assays [47,48,49,50]. A modified AAII-25 colorimetric method (Alpkem TFA analyzer; Alpkem, Clackamas, OR, USA) was used to measure serum iron and total iron-binding capacity [51,52,53]. TS was calculated by dividing serum iron concentration by total iron-binding capacity expressed as a percentage. Lastly, EPP was measured in whole blood using fluorescence extraction based on a modification of the Sassa method [45,54,55]. C-reactive protein (CRP) was measured at the University of Washington using latex-enhanced nephelometry (Dade Behring Inc., Deerfield, IL, USA) [56,57].

The following iron status indicators were used: Hb, MCV, SF, EP, SI, TIBC, TS, sTfR, and BII. We used the following thresholds to define an abnormal iron status for each biomarker shown in Table 1 below. BII stores were calculated using the formula from Cook et al. [30,31]:

BII stores (mg/kg) = −[log_10_ (sTfR × 1000 ÷ SF) − 2.8229] ÷ 0.1207.

where sTfR is soluble transferrin receptor (mg/L) and SF is ferritin (µg/L). When calculating body iron stores, we converted the Roche sTfR concentrations to those of Flowers assay equivalent [58] (Flowers = 1.5 × Roche + 0.35 mg/L) [59], as recommended in a previous comparison of the two assays [59]. We used three definitions of ID: the ferritin model [27,28], the MCV model [28,29], and the body iron model [30,31]. IDA was defined as anemia plus ID, i.e., IDA was defined as hemoglobin <12 µg/dL and at least 2 of 3 abnormal concentrations in SF (or MCV), TS, and EP or as hemoglobin <12 µg/dL and BII <0 mg/kg. Thus, participants with IDA were iron deficient and had low hemoglobin concentrations.

### 2.3. Statistical Analyses

Statistical analyses were performed using STATA (version 17; StataCorp, College Station, TX, USA) with sample weights and design variables to account for the complex sample design. The 2001–2006 NHANES 6-year examination weights were used to account for nonresponse and oversampling in all analyses. Data distributions were examined visually using kernel density plots. For ferritin, EPP, and sTfR, extreme values beyond the 1st and 99th percentiles were winsorized (replaced by the 1st and 99th percentile, respectively). Where EPP values were recorded as 0, we used ln (0.1) before analysis.

First, we calculated the arithmetic means (SEs) for serum iron, TIBC, %TS, MCV, hemoglobin, and BII. A Taylor series linearization approach was used to approximate the standard errors (SEs) for all estimates. Geometric means (95% CI) were estimated via Poisson regression [62,63] for ferritin, EP, and sTfR because their concentrations were positively skewed, as noted in previous studies [30,31,64,65]. We tested the differences in biomarker concentrations across the BMI groups using the STATA post-estimation command, lincom, after estimating the mean values.

Next, we used the svy: mean procedure with the subpopulation option to calculate the proportion of women with ID, anemia, and IDA across the BMI groups. To perform this, ID, anemia, and IDA variables were coded as 0/1. Women classified as having ID, anemia, or IDA were coded as 1 for each condition or as 0 if the condition was absent. After estimating the proportions for ID, anemia, and IDA, each module was followed immediately by running the STATA post-estimation command, lincom, to test the differences in the estimated proportions (prevalence) across the BMI groups. McNamar’s test was performed to determine if there was a statistically significant difference in the proportions of ID and IDA estimates from the ferritin, MCV, and BII models based on paired comparisons between the models.

We tested the stability of the estimates by running logistic regression models for each condition of ID, anemia, and IDA across the BMI groups, adjusting for background characteristics. ID variable, age, race and Hispanic origin, educational attainment, and family poverty-to-income ratio as independent variables were entered into the model as the dependent variables, and BMI group was entered as the independent variable. This procedure was repeated for the anemia and IDA models. We used STATA’s margins command to obtain predicted probabilities of ID, anemia, and IDA by BMI category. The differences across the BMI groups were tested using a pairwise comparison of predicted probabilities for each model. Statistical significance was set at a *p*-value of ≤0.0167 (i.e., α = 0.05/3, adjusted for the three comparisons of weight status).

## 3. Results

### 3.1. Characteristics of the Study Population

The sample comprised 2514 women with BMI data; of this, 3.0% were classified as underweight. The analyses were based on participants who were normal weight, overweight, or obese (*n* = 2442). The mean ± SD for age was 35.8 ± 6.8 years, with 43% between 20 and 34 years. The proportion categorized as normal, overweight, and obese was 42.6%, 26.2%, and 31.2%, respectively. Based on race and Hispanic ethnicity, the sample was composed of 66.7% Non-Hispanic White, 13.4% Non-Hispanic Black, 13.7% Mexican American or other Hispanic, and 5.9% of other race including multi-racial. About 43.1% of participants were from families with a family poverty-to-income ratio >350%. A quarter (26.7%) were college graduates or above.

### 3.2. Iron Biomarkers by Body Mass Index

The mean estimates of iron measures by body weight status are presented in Table 2. The mean iron status measures (SF, SI, TS, EPP, sTfR, and MCV) for this sample of women vary by BMI status. The SF (*p* < 0.001), EPP (*p* < 0.001), and sTfR (*p* = 0.005) values were higher, while those of SI (*p* < 0.001), TS (*p* < 0.001), and MCV (*p* < 0.001) were lower in women with obesity than those with normal weight. The mean estimates for EPP (*p* = 0.001) and sTfR (*p* = 0.002) were higher, while the mean estimates for SI (*p* = 0.001), TS (*p* < 0.001), and MCV (*p* < 0.001) were lower in overweight women than in normal-weight women. EPP (*p* = 0.001) and sTfR (*p* = 0.011) were lower, while SI (*p* < 0.001), TS (*p* < 0.001), and MCV (*p* < 0.001) in overweight women were higher than in women with obesity. Hemoglobin concentration was lower in women with obesity compared to women with normal weight (*p* = 0.001). The mean TIBC and BII were similar across the BMI categories. The marginal mean estimates of iron measures across the BMI categories (adjusted for age, race and Hispanic origin, educational attainment, BMI, and family poverty-to-income ratio) were also examined. Overall, there were negligible changes (0% to 2%, except for the obese category in which the adjusted estimates were 4.2% lower) in the mean estimates after adjustment for the participants’ characteristics, and the interpretation was largely unchanged. The average predicted arithmetic means ± SE for SI, TS, and MCV and the geometric means (95% CI) for SF, EPP, and sTfR vary by BMI status, similar to the unadjusted estimates. The SF (*p* = 0.019), EPP (*p* < 0.001), and sTfR (*p* < 0.001) values were higher, while those of SI (*p* < 0.001), TS (*p* < 0.001), and MCV (*p* < 0.001) were lower in women with obesity than those with normal weight. The mean hemoglobin (13.6 g/dL) was no longer different by BMI categories. The values for TIBC and BII remained similar across the BMI categories.

### 3.3. ID, Anemia, and IDA Prevalence by Body Mass Index

**Figure 1:**
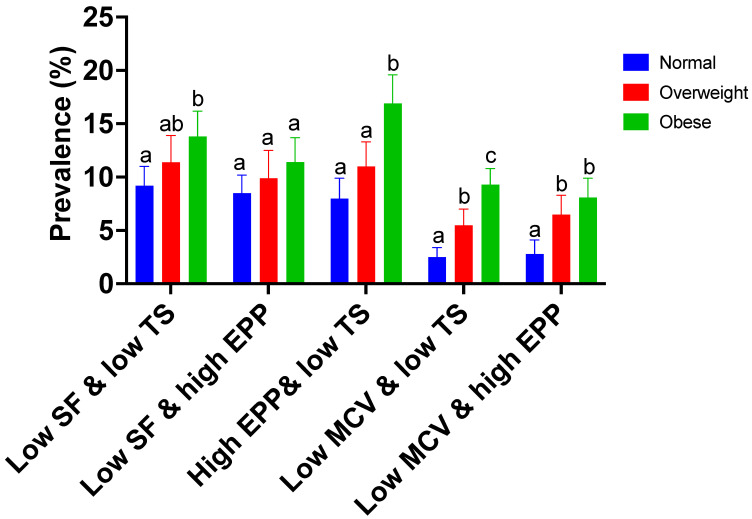
Prevalence estimates of abnormal values for two pairs of iron biomarkers are presented by BMI. NHANES, National Health and Nutrition Examination Survey; SF, serum ferritin; TIBC, total iron-binding capacity; TS, transferrin saturation; EPP, erythrocyte protoporphyrin; MCV, mean cell volume; Hb, hemoglobin; sTfR; soluble transferrin receptor. The values were calculated using the svy: mean procedure: only ID category and BMI were included in the model. ^a,b,c^ Values in the same category(e.g., Low SF and low TS) with different superscript letters are significantly different in means, *p* < 0.0167 (2-tailed *t*-test): differences in prevalence estimates were tested using the STATA post-estimation command, lincom, after estimating the mean values across the BMI categories.

Figure 1 presents the prevalence of abnormal values for two pairs of iron biomarkers. The prevalence estimates vary depending on which two combinations of abnormal iron status markers are used to define ID. ID was higher in women with obesity compared to normal-weight women when EPP was combined with TS (*p* < 0.001) but not SF (*p* = 0.094). Iron deficiency was also higher in women with obesity than those with overweight conditions based on EPP and TS (*p* = 0.001). ID was higher in women with obesity compared to normal-weight women when MCV was combined with either TS (*p* < 0.001) or EPP (*p* = 0.001). The prevalence of ID was also higher in women with obesity than in those with overweight conditions based on MCV and TS (*p* = 0.001). Deficiencies were higher across BMI categories when ferritin was combined with either TS or EPP than when MCV was combined with either TS or EPP.

The prevalence estimates of anemia, ID, and IDA are provided in Table 3. Iron deficiency estimates were lower for the BII model (8.1% to 10.9%) than the ferritin model (12.5% to 22.9%). The overall proportion (prevalence) of ID estimated from the BII model (9.6%) was lower than those estimated from the ferritin (17.1%) and MCV (13.4%) models (for all comparisons *p* < 0.001). Compared to women with normal weight, the prevalence of ID was higher in women with obesity (ferritin model: 22.9 ± 1.6% vs. 12.5 ± 1.0%, *p* < 0.001; MCV model: 20.0 ± 1.3% vs. 9.0 ± 0.9%, *p* < 0.001). The prevalence of ID was higher in women with obesity than those with overweight conditions (MCV model: 20.0 ± 1.3% vs. 12.9 ± 1.2%, *p* = 0.001). Anemia prevalence was higher in women with obesity than normal-weight women (9.3 ± 1.0% vs. 5.5 ± 0.8%, *p* = 0.002). The overall proportion (prevalence) of IDA based on the ferritin and MCV models were similar but were higher than the estimate based on the BII model (both *p* < 0.001). In women with obesity, IDA was higher than those with normal weight based on both the ferritin model (*p* = 0.001) and the MCV model (*p* = 0.001) and was just marginally higher based on the BII model (*p* = 0.011). After adjusting for age, race and Hispanic origin, educational attainment, BMI, and family poverty-to-income ratio, the ID estimates changed by ~1% point or less, and the interpretation remained unchanged.

## 4. Discussion

In the present study, we analyzed data from a large population-based cross-sectional representative dataset from the US called the NHANES. We compared several biomarkers of iron status to assess ID, anemia, and IDA across BMI categories (normal, overweight, and obese) among women aged 20–49 years. Overall, SF, EPP, and sTfR were higher in women with obesity, while SI, TS, and MCV were lower compared to normal-weight women. The estimates of ID and IDA from the BII model were lower than those obtained from the ferritin and MCV models. Based on the ferritin and MCV models but not from the BII model, ID and IDA prevalence estimates were higher in women with obesity than in normal-weight women. Anemia prevalence was marginally higher in women with obesity than those with normal weight. The results remain robust after controlling for participants’ background characteristics.

Our findings of lower serum iron and transferrin saturation in women with obesity align with reports from a systematic review showing that serum iron and transferrin saturation are lower with high BMI. This review further reported a tendency for higher ferritin concentration and lower transferrin saturation in obesity [66], which all agree with our current study results. Previous analysis of the NHANES data (1999–2006) also reported a negative association between serum iron centration and BMI in females aged 12–59 years, even after controlling for other individual characteristics [67]. Serum ferritin and soluble transferrin receptor concentrations reflect storage iron and tissue iron, respectively [24,30]. The elevated sTfR but lower SI concentration in women with obesity suggest tissue iron deficiency, despite sufficient iron stores. This is further supported by the higher level of EPP and lower %TS [68] in women with obesity compared to normal-weight women. A decreased level of serum iron in the plasma pool will result in low transferrin saturation, as observed in women with obesity. Low transferrin saturation impairs iron loading to transferrin receptors, thus depriving developing erythroblasts of an iron supply and, thereby, reducing erythropoiesis [26]. Consequently, this leads to decreased RBC synthesis, which potentially explains the resulting lower MCV values observed in women with obesity.

Furthermore, another meta-analysis reported that the odds of ID was higher in obese/overweight participants compared to normal-weight participants [21]. Although ID prevalence based on the BII model is comparable across the BMI groups, the ferritin and MCV models reveal higher ID prevalence in women with obesity compared to normal-weight women. The high prevalence of ID in women with obesity is in line with previous studies showing evidence of a strong relationship between obesity and iron deficiency in women [11,21,69,70].

These results add to the body of knowledge of population-based estimates of the prevalence of ID, anemia, and IDA that have been previously reported for different demographic characteristics using data from the NHANES 1988–1994 and 1999–2002 based on the ferritin model [71,72,73]. More recent prevalence estimates of ID have also been reported for toddlers [17], nonpregnant females [17], and pregnant females [17,32,33,74] using data from the NHANES 1999–2010; except for two studies [32,38], which reported ID estimates based on two models (ferritin and BII models), the other studies were based on the BII model. More importantly, this is the first study to simultaneously compare iron biomarkers and the prevalence of ID, anemia, and IDA across different BMI groups using the three common models for estimating population-based ID prevalence. Our results showing higher estimates of ID from the ferritin model compared to the BII model is consistent with previous findings that reported prevalence estimates for ID by comparing the ferritin model to the BII model in preschool children and nonpregnant females using data from the National Health and Nutrition Examination Survey 2003–2006 [32]. Similar to our results, the previous study also reported lower ID estimates based on the BII model (9.2%) compared to the ferritin model (15.7%) for females aged 20–49.

Although previous reports using the NHANES data have shown that ID and IDA continue to be of concern in the US, particularly in women in the reproductive age group, information on estimates across BMI groups is limited. Because iron metabolism in women with obesity is impaired compared to those without obesity [10,75,76], ID and IDA estimates by BMI for such large studies as the NHANES could provide insight into our understanding of iron status in women with obesity. This is particularly important given that more than 42% of US females aged 20+ are obese [3]. The results from our current study showed that ID and IDA prevalence estimates are high in nonpregnant women and particularly problematic in women with obesity.

The primary underlying mechanism for explaining the higher ID prevalence in women with obesity is that increased adiposity is associated with decreased duodenal iron absorption [77,78,79,80], which increases the risk of iron deficiency [11,78,81]. Iron status is primarily regulated by intestinal absorption, recycling from senescent red cells (through macrophages), and hepatocytes stored iron [82]. This systemic control is principally maintained by the hormone hepcidin, a 25-amino-acid (aa) peptide synthesized by hepatocytes [83,84], which concentration is elevated in obesity [5,6,7,85,86,87,88]. Elevated hepcidin concentration represses iron efflux from intestinal enterocytes, macrophages, and hepatocytes [89,90,91], thereby reducing iron release into the circulation and leading to low plasma iron concentration [89,92], which could lead to iron deficiency [81,93].

## 5. Conclusions

In summary, our analysis of the NHANES data revealed dysregulation of iron metabolism in women with obesity. Women with obesity had higher ferritin, EPP, and sTfR but lower SI, %TS, hemoglobin, and MCV, suggesting tissue iron deficiency (functional iron deficiency) and reduced erythropoiesis. Consequently, iron deficiency was higher in women with obesity compared to normal-weight women. The model used to classify participants as either iron deficient or not can influence the estimate of iron deficiency prevalence at the population level, particularly where obesity is common. Thus, the ID model used in defining iron deficiency should be considered when interpreting ID data or combining different studies in a meta-analysis. With the rise in severe obesity, future studies should examine how iron biomarkers and ID change across the BMI continuum, at 30 kg/m^2^ and beyond.

## Figures and Tables

**Table 1:** Cutoffs for defining abnormal values for markers of iron deficiency (SF, TS, EPP, MCV, sTfR, and BII) and hemoglobin.

Biomarkers	Abnormal Values
Serum ferritin (µg/L)	<16.5 [32]
Transferrin saturation (%)	<15 [32]
EPP (µmol/L RBC)	>1.24 [27,32]
MCV (fL)	<81 [60]
Hb (g/dL)	<12 [27]
sTfR (mg/L)	>5.3 [61]
Iron deficiency	
BII (mg/kg)	<0 [30,31]
Ferritin model	≥2 abnormal values of SF, TS, and EPP
MCV model	≥2 abnormal values of MCV, TS, and EPP

SF, serum ferritin; TIBC, total iron-binding capacity; TS, transferrin saturation; EPP, erythrocyte protoporphyrin; MCV, mean cell volume; Hb, hemoglobin; sTfR; soluble transferrin receptor.

**Table 2:** Mean ± SE (n) of iron-status measures by BMI of nonpregnant females aged 20–49 years: NHANES 2001–2006 ^1^.

	Total	Healthy Weight	Overweight	Obese
SF (µg/L) ^2,3^, µg/dL	53.5 (50.7, 56.3) (2442)	48.2 (44.8, 51.5) (899) ^a^	55.2 (48.4, 62.0) (682) ^ab^	59.4 (54.0, 64.7) (861) ^b^
SI (µg/dL)	83.1 ± 0.8 (2441)	94.3 ± 1.4 (899) ^a^	80.0 ± 1.8 (681) ^b^	70.3 ± 1.4 (861) ^c^
TIBC (µmol/L)	66.1± 0.3 (2435)	65.8 ± 0.5 (896) ^ab^	67.1 ± 0.5 (861) ^a^	65.7 ± 0.4 (858) ^b^
TS (%)	23.2 ± 0.2 (2435)	26.5 ± 0.4 (896) ^a^	22.1 ± 0.5 (681) ^b^	19.8 ± 0.5 (858) ^c^
EPP (µmol/L RBC) ^2,3^	1.05(1.03, 1.07) (2436)	0.96 (0.94, 0.99) (898) ^a^	1.06 (1.02, 1.10) (679) ^b^	1.16 (1.12, 1.21) (859) ^c^
sTfR (mg/L) ^2,3^	3.67 (3.58, 3.76) (1558)	3.37 (3.27, 3.46) (570) ^a^	3.68 (3.54, 3.82) (423) ^b^	4.05 (3.88, 4.22) (565) ^c^
MCV (fL)	89.0 ± 0.1 (2442)	90.6 ± 0.2 (899) ^a^	88.8 ± 0.3 (682) ^b^	87.2 ± 0.3 (861) ^c^
Hb (g/dL)	13.5± 0.04 (2442)	13.6 ± 0.05 (899) ^a^	13.5 ± 0.07 (682) ^ab^	13.4 ± 0.06 (861) ^b^
BII (mg/kg) ^4^	5.53 ± 0.09 (1558)	5.71 ± 0.15 (570)	5.43 ± 0.24 (423)	5. 37± 0.23 (565)

BMI, body mass index: normal weight (18.5–24.9), overweight (25.0–29.9), and obese (≥30.0). NHANES, National Health and Nutrition Examination Survey; SF, serum ferritin; TIBC, total iron-binding capacity; TS, transferrin saturation; EPP, erythrocyte protoporphyrin; MCV, mean cell volume; Hb, hemoglobin, sTfR; soluble transferrin receptor. ^1^ Values are weighted arithmetic means ± SE unless otherwise indicated. The weighted geometric means (95% CI) were calculated for SF, EPP, and sTfR. ^2^ Values beyond the 1st and 99th percentiles were winsorized (replaced by the 1st and 99th percentile, respectively). ^3^ Values are weighted geometric means (via Poisson regression) for SF, EPP, and sTfR concentrations. sTfR was collected for only two NHANES cycles (2004–2004 and 2005–2006). ^4^ Body iron index (BII) (mg/kg) = −[log10 (sTfR(mg/L) × 1000/SF (µg/L)) − 2.8229]/0.1207 [31], where sTfR and SF are soluble transferrin receptor and ferritin, respectively. sTfR concentrations were not measured for nonpregnant women for the NHANES cycle 2001–2002, and, thus, BII calculations were based on two NHANES cycles, 2003–2004 and 2005–2006. ^a,b,c^ Values in the same row with different superscript letters are significantly different, *p* < 0.0167 (2-tailed *t*-test): differences in iron-status measures were tested using the STATA post-estimation command, lincom, after estimating the mean values across the BMI groups.

**Table 3:** Prevalence (%) of ID, anemia, and IDA by BMI among nonpregnant females aged 20–49 years: NHANES 2001–2006 ^1^.

	N ^2^	Total	Healthy Weight	Overweight	Obese
ID					
Ferritin model ^3^	502	17.1 ± 0.6	12.5 ± 1.0 ^a^	17.7± 1.7 ^ab^	22.9 ± 1.6 ^b^
MCV model ^4^	407	13.4 ± 0.5	9.0 ± 0.9 ^a^	12.9 ± 1.2 ^a^	20.0 ± 1.3 ^b^
BII ^5^	181	9.6 ± 0.6	8.1 ± 1.0	10.9 ± 1.6	10.5 ± 1.2
Anemia	250	7.4 ± 0.6	5.5 ± 0.8 ^a^	8.3 ± 1.2 ^ab^	9.3 ± 1.0 ^b^
IDA					
Ferritin model ^6^	183	5.6 ± 0.5	4.1± 0.6 ^a^	6.5 ± 1.0 ^ab^	7.0 ± 0.9 ^b^
MCV model ^7^	173	5.2 ± 0.4	3.8 ± 0.6 ^a^	5.6 ± 0.8 ^ab^	6.8 ± 0.8 ^b^
BII ^8^	89	4.6 ± 0.5	3.3 ± 0.6 ^a^	5.2 ± 1.2 ^ab^	6.0 ± 1.0 ^b^

BMI, body mass index: normal weight (18.5–24.9), overweight (25.0–29.9), and obese (≥30.0). NHANES, National Health and Nutrition Examination Survey; SF, serum ferritin; TIBC, total iron-binding capacity; TS, transferrin saturation; EPP, erythrocyte protoporphyrin; MCV, mean cell volume; Hb, hemoglobin; sTfR; soluble transferrin receptor. ^1^ Values are weighted arithmetic means ± SE unless otherwise indicated. The values were calculated using the svy: mean procedure: only ID (or IDA) and BMI were included in the model. ^2^ Total unweighted samples (abnormal samples). ^3^ Defined as at least 2 of 3 abnormal concentrations in SF, TS, and EPP. ^4^ Defined as at least 2 of 3 abnormal concentrations in MCV, TS, and EPP. ^5^ ID body iron <0 mg/kg, where body iron (BII) (mg/kg) = −[log10 (sTfR(mg/L) × 1000/SF (µg/L)) − 2.8229]/0.1207 [31], where sTfR is soluble transferrin receptor and SF is ferritin. ^6^ Defined based on the ferritin model plus hemoglobin <12 µg/dL. ^7^ Defined based on the MCV model plus hemoglobin <12µg/dL. ^8^ Defined based on the BII model plus hemoglobin <12 µg/dL. Abnormal values for ferritin, TS, EPP, MCV, sTfR, hemoglobin, and BII are 16.5 µg/L, <15%, >1.24 µmol/L RBC, <81fL, 5.3 mg/L, <12µg/dL, and BII <0 mg/kg, respectively. ^a,b^ Values in the same row with different superscript letters are significantly different in means, *p* < 0.0167 (2-tailed *t*-test): differences in prevalence estimates were tested using the STATA post-estimation command, lincom, after estimating the mean values across the BMI categories.

## Data Availability

NHANES data are publicly available from the CDC website at https://www.cdc.gov/nchs/nhanes/index.htm (accessed on 18 August 2021).
